# Rapid Microbial Community Changes During Initial Stages of Pine Litter Decomposition

**DOI:** 10.1007/s00248-018-1209-x

**Published:** 2018-05-30

**Authors:** Marcin Gołębiewski, Agata Tarasek, Marcin Sikora, Edyta Deja-Sikora, Andrzej Tretyn, Maria Niklińska

**Affiliations:** 1 Nicolaus Copernicus University, Lwowska 1, 87-100 Toruń, Poland; 2 Centre for Modern Interdisciplinary Technologies, Nicolaus Copernicus University, Toruń, Poland; 3 Institute of Environmental Sciences, Jagiellonian University, Kraków, Poland; 4 Department of Microbiology, Nicolaus Copernicus University, Toruń, Poland

**Keywords:** Pine litter decomposition, Bacterial community, Fungal community, Metagenomics, 16S rDNA pyrosequencing

## Abstract

Plant litter decomposition is a process enabling biogeochemical cycles closing in ecosystems, and decomposition in forests constitutes the largest part of this process taking place in terrestrial biomes. Microbial communities during litter decomposition were studied mainly with low-throughput techniques not allowing detailed insight, particularly into coniferous litter, as it is more difficult to obtain high quality DNA required for analyses. Motivated by these problems, we analyzed archaeal, bacterial, and eukaryotic communities at three decomposition stages: fresh, 3- and 8-month-old litter by 16/18S rDNA pyrosequencing, aiming at detailed insight into early stages of pine litter decomposition. Archaea were absent from our libraries. Bacterial and eukaryotic diversity was greatest in 8-month-old litter and the same applied to bacterial and fungal rDNA content. Community structure was different at various stages of decomposition, and phyllospheric organisms (bacteria: Acetobacteraceae and Pseudomonadaceae members, fungi: *Lophodermium*, *Phoma*) were replaced by communities with metabolic capabilities adapted to the particular stage of decomposition. Sphingomonadaceae and Xanthomonadaceae and fungal genera *Sistotrema*, *Ceuthospora*, and *Athelia* were characteristic for 3-month-old samples, while 8-month-old ones were characterized by Bradyrhizobiaceae and nematodes (*Plectus*). We suggest that bacterial and eukaryotic decomposer communities change at different stages of pine litter decomposition in a way similar to that in broadleaf litter. Interactions between bacteria and eukaryotes appear to be one of the key drivers of microbial community structure.

**Electronic supplementary material:**

The online version of this article (10.1007/s00248-018-1209-x) contains supplementary material, which is available to authorized users.

## Introduction

Decomposition of organic matter is a key step in nutrients cycling in all ecosystems. Plant litter decomposition is an important ecological process enabling biogeochemical cycles closing in terrestrial ecosystems. Decomposition in forests constitutes the largest part of this process taking place in terrestrial biomes, due to their immense area (~ 30% of land surface [1]) and large quantities of organic matter stored.

Forest litter decomposition was extensively studied since the early 1970s (reviewed, e.g., in [2]). However, chemical changes in decomposing materials as well as element cycling in relation to temperature and precipitation were assessed, while microbial and macroorganismal aspects of this process were less intensively studied [3, 4]. Nevertheless, it is estimated that microbes are responsible for up to 90% of organic matter decomposition [5], and the dominating primary decomposers in boreal and temperate forest soil systems are microorganisms, mainly fungi and bacteria. The structure and development of decomposer communities can influence the pattern of decay [3].

As the material decomposes, chemical composition of the litter changes, and there is a shift from carbohydrates and aliphatic components constituting the largest pool in initial litter, to aromatic compounds at late stages of decomposition. Many components of fresh plant litter, like sugars and peptides, decompose quickly, as they are energy-rich and can be easily assimilated by soil microorganisms [6–11]. The chemical changes coupled with mixing the original plant substrate with soil particles, due to the action of annelids and arthropods, enable supporting different decomposer communities.

Microbial communities at various stages of litter decomposition were studied mostly with traditional microbiological methods [12–14] and more recently using molecular approach [15–20]; however, the studies mainly concerned broad-leaf forest litter [17, 21, 22]. Coniferous substrates rich in waxes, resins, and lignin are more resistant to decomposition [23, 24] and more difficult to study; thus, only a few studies were performed to date [25, 26]. Nevertheless, a general picture of microbial succession on litter was obtained, in which phyllospheric organisms, such as members of Acetobacteraceae among bacteria and Leotiomycetes among fungi act as early decomposers [27] and are quickly replaced with distinct communities characteristic for particular stages of decomposition [17, 19]. Fungi seem to be the key decomposers responsible for producing extracellular hydrolytic and oxidating enzymes, and among them, Ascomycota prevail at early stages of decomposition [16] and are replaced by Basidiomycota later on [17, 28]. Bacterial communities tend to be dominated by Proteobacteria, Bacteroidetes, and Actinobacteria [19, 20]; however, Acidobacteria were found to be frequent in spruce litter [26]. It was found that both bacterial and fungal diversity generally increased in the process of decomposition [17, 19]. However, as decomposition spans many years, one has to bear in mind that seasonality is also an important factor shaping litter microbial community structure [26].

Until fairly recently, microbes were studied with the use of culture-based methods, which limited the scope of the studies to culturable organisms. Usually less than 1% of microbes from a given environment can be cultured under laboratory conditions [29–31]. A set of methods was devised, collectively termed “metagenomics” [32], which allows overcoming of the culturability problem. It is based on isolation of genetic material directly from the environment, without prior culturing of microorganisms [33]. A pre-requisite for all of them is DNA isolation directly from environmental sample. There are many DNA isolation methods successfully applied to soil samples, but there is only a couple of examples of DNA isolation from forest litter [34]; moreover, they concern mostly broadleaf forest litter [19], which has different chemical properties, with less phenolics and other compounds that could possibly affect DNA extraction efficacy and hamper subsequent molecular analyses [35]. Standard methodology of environmental DNA isolation now includes the use of bead beating-based kits. We wanted to check if combining a commercial kit with enzymatic digestion with lysozyme, achromopeptidase, and chitinase would improve the yield of DNA and spectrum of lysed organisms. We hypothesized that the treatment would increase the yield and diversity of 16/18S rDNA amplicons. As there were no reports of Archaea being found in litter, we expected that they would be absent from pine litter samples. Bacterial and eukaryotic communities would be different at different stages of decomposition, specifically (i) microbial diversity would follow diversity of substrates, i.e., would be lower at later stages of decomposition, (ii) late communities would be more dominated by specialists capable of lignocellulose and lignin utilization, (iii) phyllosphere-related organisms would prevail initially and then would be replaced by those coming from soil, and (iv) a shift in metabolic capabilities of the community was also expected wherein organisms utilizing soluble small molecules initially present in the phyllosphere would be superseded by those in whose genomes reside genes enabling cellulose and, later, lignin utilization. To test these hypotheses, we prepared, sequenced, and analyzed pyrosequencing (454) libraries of archaeal and bacterial 16S, as well as eukaryotic 18S rRNA gene fragments derived from litter samples at three stages of decomposition.

## Materials and Methods

### Study Design and Samples

The study site was located in Sierbowice, southern Poland (GPS coordinates: N 50° 34′ 15.20″, E19° 39′ 55.40″). The vegetation at the site is dominated by approx. 50 years old Scots pine (*Pinus sylvestris* L.) with a small admixture of birch (*Betula pendula* Roth) and artificially introduced red oak (*Quercus rubra* L.), with European blueberry (*Vaccinium myrtillus* L.) and mosses in the groundcover.

We were interested in investigating three time points of initial stages of litter decomposition: *t*_0_ = no field incubation, *t*_1_ = 92 days, and *t*_2_ = 242 days of field incubation, because we expected changes in community composition in line with rapid initial mass loss. In order to do so, freshly fallen brown pine litter from over a dozen of randomly picked trees in the area of about 3 ha was collected in October 2012. The needles were obtained by shaking the selected trees and their branches. A PVC foil was spread under the trees to facilitate litter collection and to prevent contamination of fresh litter with microbial communities from soil.

The collected litter was transported to the laboratory in plastic bags. The material was thoroughly mixed and split into two major parts. The first part consisted of three subsamples dedicated for water content analysis and DNA extractions to determine the *t*_0_ microbial community. The second major part of the collected material was air dried in room temperature for a month. Three subsamples from the air-dried material were taken for chemical analyses, and the rest was used to make litter bags. The litter bags were made out of a nylon mesh (20 × 20 cm, mesh size 1 × 1 mm) and were packed with ~ 20 g of air dry litter. On the 1 December 2012, they were placed back in the field under the soil organic layer. Six bags were collected at each of the time points. Every time, three litter bags were dedicated for chemical and the remaining ones for molecular analyses. Before the DNA extractions, the samples were placed in a climatic chamber and acclimated for a week at 22 °C to 70% of water holding capacity (WHC) and frozen at − 80 °C. The acclimation step was performed in order to standardize the physiological state of microorganisms in the litter, as the collection of litter took place during different seasons. In the end, we obtained three biological replicates per time point, and three technical replicates (independent isolations) were made for each of four isolation methods used. Just as in case of the *t*_0_ series before the chemical analyses, *t*_1_ and *t*_2_ samples were air dried.

### Physicochemical Analyses

Water content was measured gravimetrically in fresh litter samples, immediately after their transfer to the laboratory. pH was measured in a slurry of 0.5-g air dried litter in 15 ml of demineralized water. The concentrations of particular elements were measured in powdered and dried material (12 h, 105 °C). C and N concentrations were determined with Vario EL Cube (Elementar, Germany). The remaining macroelements and microelements were determined with a PinAAcle 900 Z atomic absorption spectrometer (Perkin Elmer, USA) after wet digestion with nitric acid in Titan MPS microwave sample preparation system (Perkin Elmer, USA).

### Climatic Data

Climatic data (daily averages) were downloaded from dane.imgw.pl. As there is no meteorological station in the immediate vicinity of the sampling area, values from six nearest stations (Silniczka, Lgota Górna, Katowice-Pyrzowice, Olewin, Miechów, Jędrzejów-Sudół) were averaged and plotted in R.

### DNA Isolation

PowerSoil DNA Isolation kit (MoBio, USA) was used according to the producer’s protocol for PowerLyzer 24 bead beater involving one 45-s cycle of bead beating at 4000 rpm for control isolations and with the following modifications: (i) addition of lysozyme (Sigma, USA) to the final concentration of 2 mg/ml and achromopeptidase (500 U/ml, Sigma, USA) to the C1 buffer and incubation at 37 °C for 1 h (AL method), (ii) addition of chitinase (Sigma, 0.01 U/ml) and incubation at for 1 h (Ch method) and (iii) addition of lisozyme, achromopeptidase and chitinase, and incubation as above (ALCh method). Isolated DNA was quantified with Qubit HS DNA kit (Invitrogen, USA), and the quality was measured spectrofotometrically on NanoDrop ND-1000 (Thermo Fisher Scientific, USA). DNA content was expressed in ng per 1 g of fresh litter.

### Primers Design

Primers were designed basing on SILVA v.119 alignment [36]. The alignment was split into kingdom-specific parts with Mothur’s get.lineage, and consensus sequences were generated at the 97% identity level (consensus.seqs). Visual inspection of resulting summary files allowed identification of highly conserved regions. Candidate pairs were checked with the online TestPrime tool [37] and IDT Oligoanalyzer [38]. Pine-specific primers were designed in the same way, but an alignment of *Pinus* 18S rRNA sequences from SILVA was used.

### 16S and 18S rRNA Gene Fragments Amplification and Pyrosequencing

Libraries of 16S/18S rRNA gene fragments were created with the use of two-step method, involving gene-specific primers tagged with M13/M13R overhangs in the first round of PCR and M13 bearing 9-nt MID sequences [39] and A adapter sequence (Roche, Switzerland) paired with M13R with B adapter overhang in the second round. Primer sequences and PCR conditions are listed in Table 1. The final products were quantified with Qubit HS DNA kit (Invitrogen), and 36 of them were pooled in equimolar amounts for each library. The library quality was assessed with HS DNA chip on Bioanalyzer (Agilent, USA). Libraries were emPCR amplified with the use of Titanium Lib-L kits (Roche, Switzerland) and sequenced on GS-Junior machine with Titanium chemistry (Roche, Switzerland) as per the manufacturer’s protocols.

**Table 1:** Primer sequences and PCR conditions

Name^a^	Sequence 5′ → 3′^b^	Paired with	Target	PCR conditions	Use	Source
B357fU	**GTTTTCCCAGTCACGAC** CCT ACG GGA GGC AGC AG	B786rU	Bacterial 16S	95 °C–5 min; 25 cycles of 95 °C–15 s, 53 °C–30 s, 72 °C–30 s	Library generation	Neefs, 1993 [81]
B786rU	**CAGGAAACAGCTATGAC** AC CAG GGT ATC TAA WCC	B357fU				Deja-Sikora, 2012 [61]
A519fU	**GTTTTCCCAGTCACGAC** CAG CMG CCG CGG TAA	A1048rU	Archaeal 16S	95 °C–5 min; 25 cycles of 95 °C–15 s, 51 °C–30 s, 72 °C–30 s	Library generation	Klindworth, 2013 [37]
A1048rU	**CAGGAAACAGCTATGAC** CGR CRG CCA TGY ACC WC	A519fU				
E566fU	**GTTTTCCCAGTCACGAC** CAG CAG CCG CGG TAA TTC C	E1200rU	Eukaryotic18s	95 °C–10 min; 25 cycles of 95 °C–15 s, 53 °C–30 s, 72 °C–30 s	Library generation	Hadziavdic, 2014 [82]
E1200rU	**CAGGAAACAGCTATGAC** CCC GTG TTG AGT CAA ATT AAG C	E566fU				
M13-x-A^c, d^	CCA TCT CAT CCC TGC GTG TCT CCG AC *TCAG* X **GTT TTC CCA GTC ACG AC**	M13R-B	M13-tagged amplicons	95 °C–5 min; 10 cycles of 95 °C–15 s, 52 °C–45 s, 72 °C–45f s	Library generation	This work
M13R-B^c^	CCT ATC CCC TGT GTG CCT TGG CAG TC *TCAG* **CAG GAA ACA GCT ATG AC**	M13-x-A				
B969f	ACG CGA RGA ACC TTA C	B1072r	Bacterial 16S	95 °C–10 min; 40 cycles of 95 °C–15 s, 53 °C–30 s, 72 °C–30 s	qPCR	
B1072r	CGA GCT GAC GAC ARC CAT GCA	B969f				
ITS1	TCC GTA GGT GAA CCT GCG G	qITS2	Fungal ITS	95 °C–10 min; 40 cycles of 95 °C–15 s, 55 °C–30 s, 72 °C–30 s		White, 1990 [79]
qITS2	TTY GCT GYG TTC TTC ATC G	ITS1				Wakelin, 2007 [80]
Con1256f	TTA TTC CTG GTT CGA GA	Pin1360r	Coniferae 18S	95 °C–10 min; 40 cycles of 95 °C–15 s, 49 °C–30 s, 72 °C–30 s		This work
Pin1360r	TAG TCA ACA CGA GTT GA	Con1256f	Pinus 18S			

^a^U denotes M13/M13R tagged sequences

^b^M13 and M13R sequences are given in boldface font

^c^Key sequence in italics

^d^X denotes 10-nt barcode (MID sequence), for all barcode pairs Levenstein distance was min. 4

### qPCR

Real-time PCR analyses were conducted using primers listed in Table 1 and FastStart SYBRGreen kit (Roche, Switzerland) on LightCycler 480 machine (Roche, Switzerland). The reaction mix included 3 pmol of forward and reverse primers, 2 ng of template DNA, 5 μl of 2 x concentrated kit, and water up to 10 μl. Standards were prepared from pure amplicons generated with primer pairs used for qPCR on DNA isolated from *Escherichia coli*, *P. sylvestris*, and *Boletus badius*. Standard curves were replicated five times, and samples were assayed in triplicates. Each run included negative control (water). Resulting numbers of copies were converted to copies/g of litter.

### Bioinformatics Analyses

Pyrosequencing reads were processed with Mothur v. 1.32 [40] and custom-tailored Perl scripts, with modifications increasing the aggressiveness of denoising, chimera removal, as well as producing ten subsamples of the whole data and averaging the shared OTU table over those subsamples, as described earlier [41, 42]. Brief summary of the procedure is given below.

The flows were extracted from the .sff files, forward, and reverse reads separately (sffinfo), then they were assigned to samples basing on the MID sequences, trimmed to min. 500 and max. 650 flows (trim.flows), and denoised with AmpliconNoise algorithms (shhh.flows and shhh.seqs; [43]). Primers and MIDs were removed from the denoised seuqences, and the read set was dereplicated (unique.seqs) and aligned to the SILVA v.119 template alignment (align.seqs). Reads covering the desired region of the alignment (pos. 6500–22,500 for bacteria and 13,876–22,550 for eukaryotes) were chosen (screen.seqs) and gap only, and terminal gap-containing columns were removed from the alignment (filter.seqs). The set was dereplicated again, and residual sequencing and PCR noise was removed with Single Linkage pre-clustering (pre.cluster; [44]). Chimera identification and removal were performed in two rounds: (i) with UCHIME (chimera.uchime; [45]) and (ii) with PERSEUS (chimera.perseus; [43]).

Full-length sequences (list.seqs, get.seqs) were used for classification with naive Bayesian classifier (classify.seqs; [46]) using SILVA 119 template and taxonomy files (http://www.mothur.org/w/images/2/27/Silva.nr_v119.tgz, accessed on September 4, 2014) for classification of bacterial reads and PR2 database [47] for eukaryotic ones at the bootstrap confidence level of 80%. Taxons “unknown” and, in case of bacterial data, “chloroplast” were removed from the final set. OTUs at the 0.03 dissimilarity level were constructed via average linkage (UPGMA), and singletons together with doubletons were removed from the data (remove.rare).

For the initial analyses of enzyme influence, the final read set was subsampled to 500 reads per sample ten times (sub.sample and regular expressions in the sed editor), subsamples were combined (cat), the whole set was dereplicated and used for distance matrix calculation (dist.seqs), and OTU construction via average neighbor clustering at 97% similarity level (cluster). Shared OTU table was constructed (make.shared), and averaged table was calculated with a Perl script (average_shared.perl). Final analyses were performed on a dataset in which all enzymatic treatments coming from one sample were combined. This dataset was subsampled to 700 (bacteria) and 1000 (eukaryota) reads per sample and processed as described above.

Relaxed neighbor joining (RNJ) tree was constructed from the final alignment with clearcut (clearcut; [48]). UniFrac [49] distance matrices were calculated in Mothur (unifrac.unweighted and unifrac.weighted) with subsampling the RNJ tree to include 700 and 1000 reads per sample for bacteria and eukaryota, respectively. Morisita-Horn [50] and Bray-Curtis [51] distance matrices were calculated in R (vegdist). NMDS and CCA analyses were performed in R with vegan’s [52] metaMDS and cca functions, respectively. For NMDS, 1000 tries were used, and the same number of permutations was adopted in CCA. CCA models were built by backward selection with ordistep.

Co-correspondence analysis [53] was performed with the use of the coca function from the cocorresp R package [54]. Significance of the extracted axes was tested with permutation test (permutest), while the percent of fit was checked by leave-one-out cross-validation (crossval).

### PICRUSt Analysis

For PICRUSt [55] analysis, bacterial sequences were classified as described above, but using GreenGenes taxonomy files (v. 13_8; [56]). Taxonomic information along with OTU table was converted to a .biom file using Mothur’s make.biom function. The file was then converted to v.1.0.0 format using biom convert, as per https://github.com/rprops/PICRUSt_from_mothur (visited Feb. 15, 2017). Normalized OTU table was generated with normalize_by_copy_number.py, and predicted metagenomes as well as NSTI scores were calculated with predict_metagenomes.py. Functions were collapsed to pathways at level 2 using categorize_by_function.py. Pathways pertaining to organismal systems were considered spurious and removed. Predicted metagenomes were analyzed with STAMP [57].

### Statistical Analyses

R [58] was used for statistical computations, with Hmisc [59], phyloseq [60] and vegan [52] packages. Significance level used was 0.01. ANOVA with Tukey’s HSD test was used to test for significance of differences in means, PERMANOVA (vegan’s adonis), AMOVA (amova of ade4 package), as well as ANOSIM (vegan’s anosim) for testing separation of clusters. Significance level used was 0.01. When testing for significance of grouping by enzymatic treatments, permutations were restricted to a given decomposition level (strata = decomposition). PERMDISP test (vegan’s betadisper) was used to test the homogeneity of variance in community data.

Differences in KEGG pathway content between litter decomposition stages were tested in STAMP using Kruskal-Wallis test with two-sided Welch’s test as a post hoc analysis and Benjamini-Hochberg FDR correction, which was required due to the non-normal distribution of *P* values. *Q* value threshold of 0.05 was assumed. Significance of sample clusters separation was tested with AMOVA on a Bray-Curtis distance matrix derived from the simulated metagenome count table (vegan’s vegdist). Functional diversity was calculated as the number of categories at level 0, which is the level of individual Kegg Orthologies (might be understood as functions).

Species diversity was measured as Shannon’s diversity (*H′*), species richness was measured as observed number of OTUs, and evenness was estimated as Shannon’s evennes (*J′*). From now on, for the sake of brevity, we will use terms “diversity” and “evenness” in place of their indices names.

## Results

### Changes of Climatic and Litter Physicochemical Variables over Time

Average daily temperature at the sampling area was below 0 °C for the majority of the period preceding *t*_1_, rose to around 12 °C 3 weeks later, and then after a month it fluctuated around 15 °C until the end of the experiment. Precipitation was low (1.2 mm daily on average) until day 150; in this period, it was in the form of snow, but the snow cover lasted until day 140. Later, rainfalls were more intensive (3.8 mm daily) until day 230, after which a 3-week period of drought occurred. Final sampling was performed 2 weeks after the onset of drought (Supplementary Fig. 1).

C/N ratio as well as K level were the highest in *t*_0_ samples and stayed at lower level in older ones (Table 2). An increasing trend was apparent for Mg, Fe, and Zn, while concentration of Mn was the highest in *t*_1_ and Cu in *t*_2_ samples. pH was similar in all samples; the average was 5.22 ± 0.37. Water content of freshly fallen litter (*t*_0_ samples) was ~ 27%, while directly after removal from the field, *t*_1_ was significantly more humid and contained 53% water and humidity in *t*_2_ was even lower than in *t*_0_ (23%).

**Table 2:** Mean values of C/N ratio and concentrations of other elements as well as water content in litter samples. Significant differences (ANOVA with Tukey’s HSD, *P* < 0.01) are denoted with different letters

Code	time	C/N	Mn^a^	Mg^a^	K^a^	Cu^a^	Fe^a^	Zn^a^	WC (%)
*t*0	0	64.7A	665.7A	434.0A	3061.6A	3.3A	288.1A	125.3A	27
*t*1	92	48.4B	721.9B	644.1B	2659.9B	3.0A	330.4B	136.7B	53
*t*2	242	47.6B	601.3C	820.3C	2388.2B	5.8B	530.8C	189.7C	23

^a^mg-kg^−1^ dry weight

### Archea Are Absent from Litter Samples

No archaeal reads were recovered during the project. The sequences coming from the libraries prepared using the presumed archaeal primers [37] were classified either as Bacteria (majority as Actinobacteria) or as Eukaryotes (Fungi). As the primers have perfect matches to almost 80% of archaeal sequences in Silva, we think that no Archaea, at least such recoverable with the primer pair used, were present in samples under study.

### Addition of Enzymes Does Not Improve DNA Isolation Yield and Microbial Diversity Recovered

**Fig. 1:**
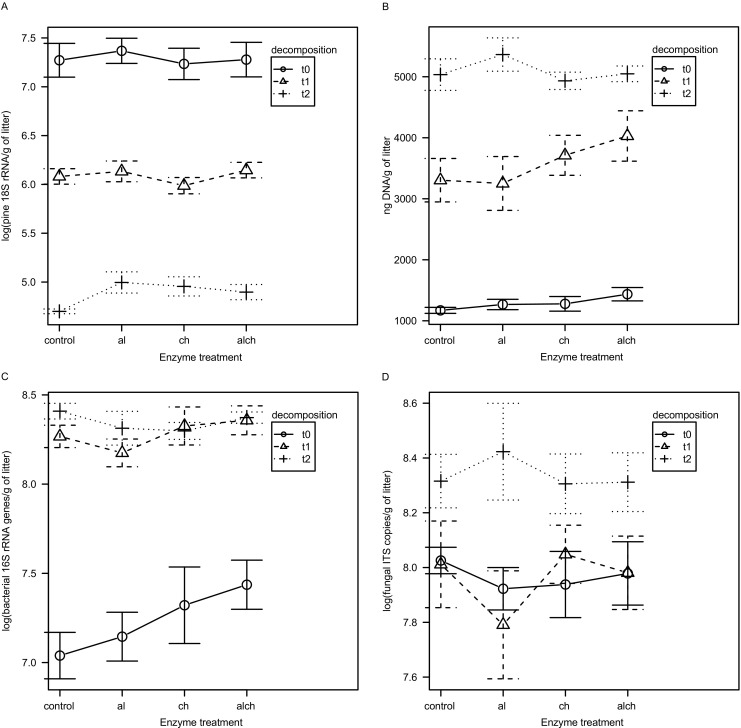
Influence of DNA isolation method modifications on: the number of pine 18S rRNA genes (**a**), overall DNA yield (**b**), the number of bacterial 16S rRNA genes (**c**), the number of fungal ITS sequences (**d**). Note the logarithmic y axis in panels **a**, **c**, and **d**. Whiskers denote standard error of the mean (SEM). Control—no enzymatic treatment, al—achromopeptidase + lysozyme, ch—chitinase, alch—achromopeptidase + lysozyme + chitinase

Pre-treatment of litter samples with achromopeptidase and lysozyme, chitinase, and all three enzymes together did not affect the copy number of pine 18S rRNA gene in litter metagenomic DNA (*P* > 0.05, Fig. 1a). The overall DNA yield was not changed by the addition of enzymes (*P* > 0.05, Fig. 1b). The same applies to the number of copies of bacterial 16S rRNA genes measured with qPCR (*P* > 0.05, Fig. 1c) and the number of copies of fungal ITS sequences (*P* > 0.05, Fig. 1d).

**Fig. 2:**
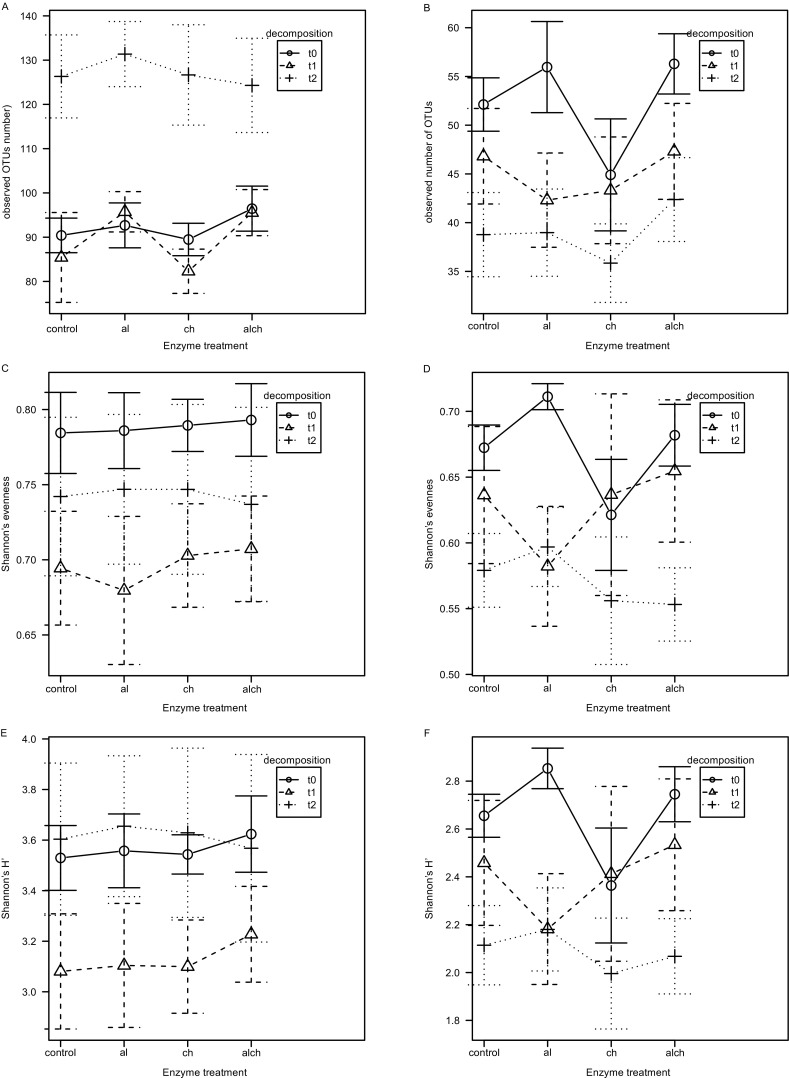
Influence of DNA isolation method modifications on: bacterial (**a**) and eukaryotic (**b**) species richness (observed number of OTUs), bacterial (**c**) and eukaryotic (**d**) Shannon’s evenness, and bacterial (**e**) and eukaryotic (**f**) Shannon’s diversity. Whiskers denote standard error of the mean (SEM). Control—no enzymatic treatment, al—achromopeptidase + lysozyme, ch—chitinase, alch—achromopeptidase + lysozyme + chitinase

Bacterial and eukaryotic diversity (measured as Shannon’s *H′*), species richness as well as evenness were not influenced by modifications of the DNA isolation method, but they differed at various stages of decomposition (Fig. 2). The same applies to community structure, as assessed with AMOVA and ANOSIM performed on Bray-Curtis and Morisita-Horn distance matrices (*P* > 0.05; data not shown).

### DNA Content and Bacterial as well as Fungal SSU Gene Copy Numbers Change During Early Stages of Decomposition

**Fig. 3:**
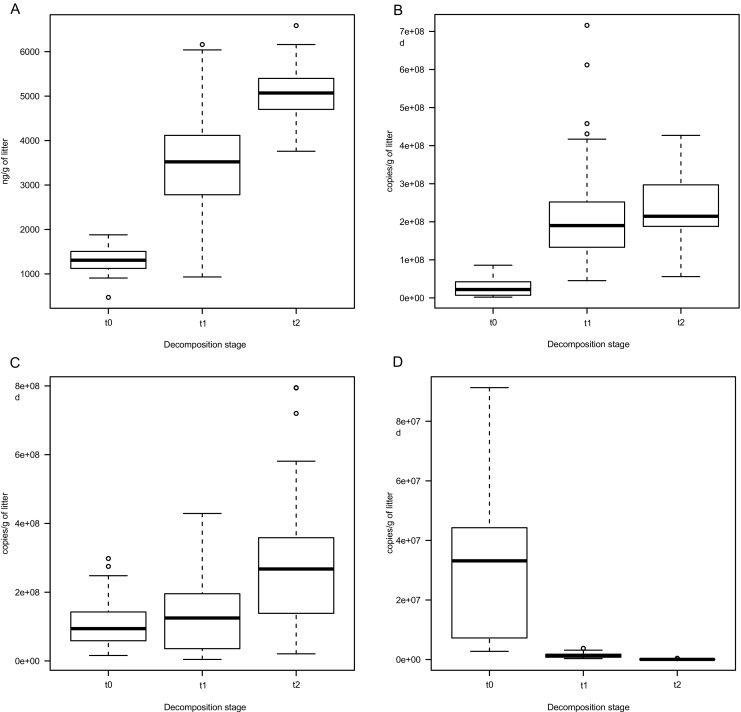
Changes in DNA composition and yield during decomposition. DNA yield (**a**), number of bacterial 16S rRNA genes (**b**), number of fungal ITS sequences (**c**), number of pine 18S rRNA genes (**d**). Whiskers denote standard error of the mean (SEM)

DNA yield significantly increased over time (*P* < 0.05, Fig. 3a). A rapid decrease of pine 18S rRNA gene copy number in *t*_1_ and *t*_2_ samples was observed (*P* < 0.05), indicating that most of pine DNA was degraded after 3 months (Fig. 3d). Thus, it seemed that the increase in yield was due to the higher bacterial and fungal cell numbers. Indeed, the copy numbers of both bacteria 16S rRNA genes and fungal ITS sequences, being proxies for bacterial and fungal cell numbers, were greatest in *t*_2_ samples. However, the number of bacterial SSU was significantly higher in *t*_1_ than in *t*_0_ (Fig. 3b), while fungal ITS number was significantly higher in *t*_2_ samples than in *t*_1_ (Fig. 3c).

### Microbial Diversity Changes During Litter Decomposition

**Fig. 4:**
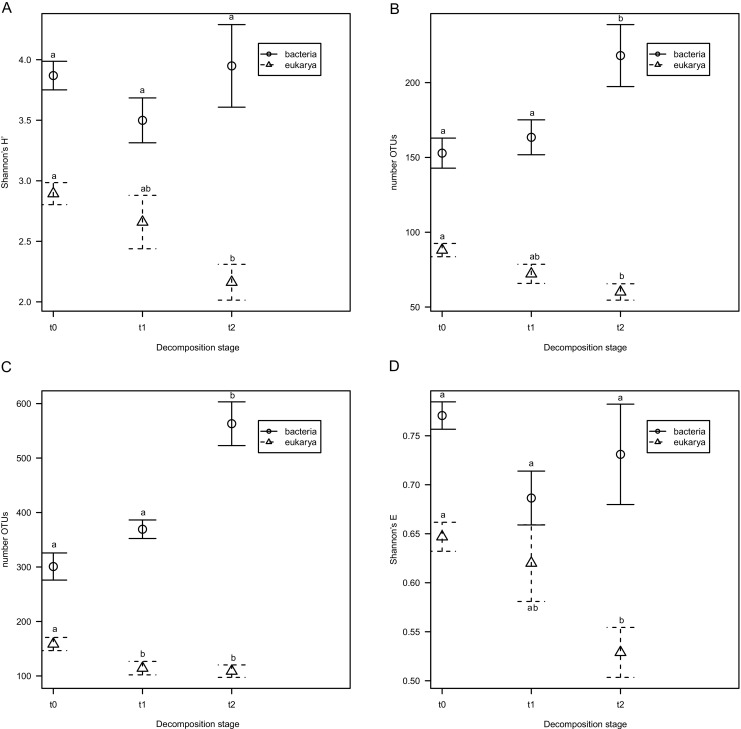
Changes in species richness, evennes and diversity estimates. Shannon’s *H′* for bacterial (**a**), observed number of OTUs (**b**), Chao1 estimated total number of OTUs (**c**), Shannon’s evenness (**d**)

Bacterial diversity (measured as Shannon’s *H′*) was similar in all samples; it was highest in fresh litter, then dropped after 3 months to reach higher values again after 8 months; however, these changes were not significant (Fig. 4a). On the other hand, eukaryotic diversity decreased rapidly over time (Fig. 4a), and the difference between fresh and 8 months old litter was significant. Both observed and estimated total (Chao1) species richness grew with time in the case of bacteria and decreased in the case of eukaryotes (Fig. 4, b, c), while evenness followed the pattern of diversity in both cases (Fig. 4d).

### Bacterial and Fungal Community Structure Is Driven by Decomposition Stage, Physicochemical Variables, and Bacteria-Eukaryote Interactions

**Fig. 5:**
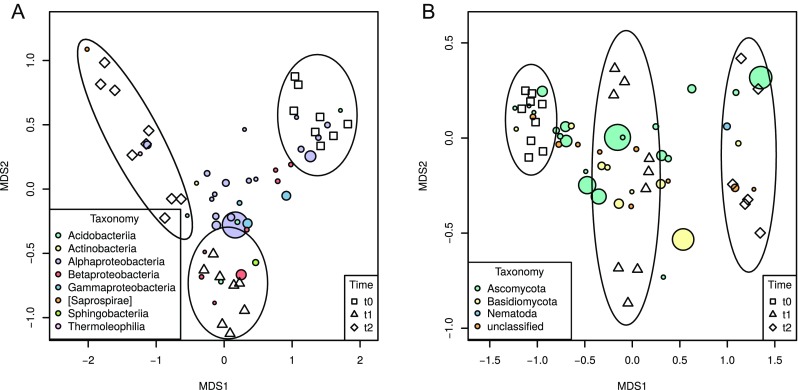
Non-metric multidimensional scaling (nMDS) analysis of Bray-Curtis distance matrices for bacterial (**a**) and eukaryotic (**b**) communities. Stress: bacteria—0.085, eukaryota—0.099

Regardless of the dissimilarity measure used, bacterial communities coming from individual biological replicates clustered tightly on nMDS plots, and samples at the same stage of decomposition were also located together (Bray-Curtis showed on Fig. 5a). It appeared that the *t*_1_ samples were more similar to the fresh ones (*t*_0_) than to the *t*_2_ samples. On the other hand, eukaryotic communities in *t*_1_ samples were closer to *t*_2_ ones (Bray-Curtis showed on Fig. 5b). ANOSIM as well as AMOVA and PERMANOVA analyses showed that separation of clusters was significant (*P* < 0.05). PERMDISP test demonstrated that variance was not homogeneous in different sample groups for bacterial community, with highest dispersion found in *t*_2_ samples (*P* < 0.05), while it was similar in all groups for eukaryotic community (*P* = 0.848).

CCA analysis (Supplementary Fig. 2) identified Zn and Na as significant environmental parameters shaping the bacterial community structure (*P* < 0.01), while C/N ratio as well as Zn and Mn were factors that significantly influenced eukaryotic community. The measured variables explained 20.1% of inertia (variance) in case of bacteria and 37.2% in case of eukaryotes.

Possible influence of eukaryotes on bacterial community and vice versa was assessed with co-correspondence analysis (Supplementary Fig. 3). Two first COCA axes turned out to be significant in a permutation test (*P* < 0.05), and they were sufficient (i.e. adding more axes would not significantly increase percent variance explained) according to leave-one-out cross-validation. They explained 22.4% of variance in the bacterial community and 44.15% of variance in the eukaryotic community. Mean distance between bacterial and eukaryotic site coordinates was significantly higher for *t*_2_ samples (0.985, *P* < 0.05), than for *t*_0_ (0.673) and *t*_1_ (0.646) ones, indicating that stronger influence was exerted in the latter two sample groups.

### Bacterial Community in Litter Samples Is Dominated by Proteobacteria

**Fig. 6:**
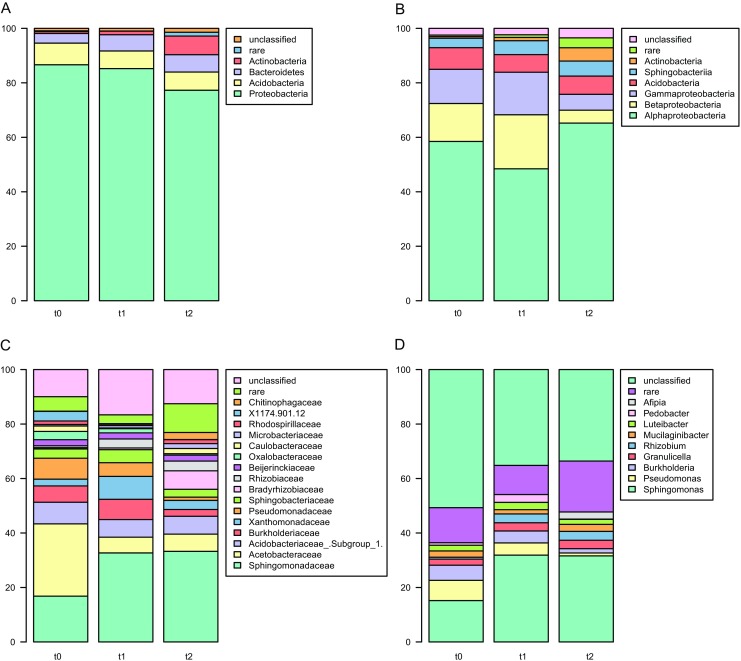
Classification of bacterial reads. Percentages of all reads are shown at the following levels: phyla (**a**), classes (**b**), families (**c**), and genera (**d**)

Although bacterial community structure varied greatly in biological replicates, certain general trends were visible. At all levels an increase of rare taxa levels in time was found, which was in line with increasing bacterial diversity. At the level of phylum, the community was dominated by Proteobacteria (68–88%), and an increase of Actinobacteria was visible in *t*_2_ samples (Fig. 6a). Only four phyla (Proteobacteria, Acidobacteria, Bacteroidetes, and Actinobacteria) accounted for the vast majority of reads in all samples. Among classes Alphaproteobacteria dominated in all libraries, and slight decrease of Beta- and Gammaproteobacteria in *t*_1_ and *t*_2_ samples was observed with concomitant increase of Actinobacteria, Sphingobacteriia, and rare taxa levels (Fig. 6b). More changes were visible at the order level, e.g., Rhodospirillales reads were abundant in *t*_0_ reads and significantly less numerous in *t*_1_ and *t*_2_, Burkholderiales and Xanthomonadales were most frequent in *t*_1_ samples, while Rhizobiales and Sphingobacteriales reads were most frequent in *t*_2_. The same pattern was observed for families within the abovementioned orders (Fig. 6c) and among genera, where *Sphingomonas* was the most frequent one, and *Pseudomonas*, *Burkholderia*, *Granulicella*, and *Rhizobium* were also found in large quantities (Fig. 6d).

### Eukaryotic Community in Litter Samples Is Dominated by Fungi

**Fig. 7:**
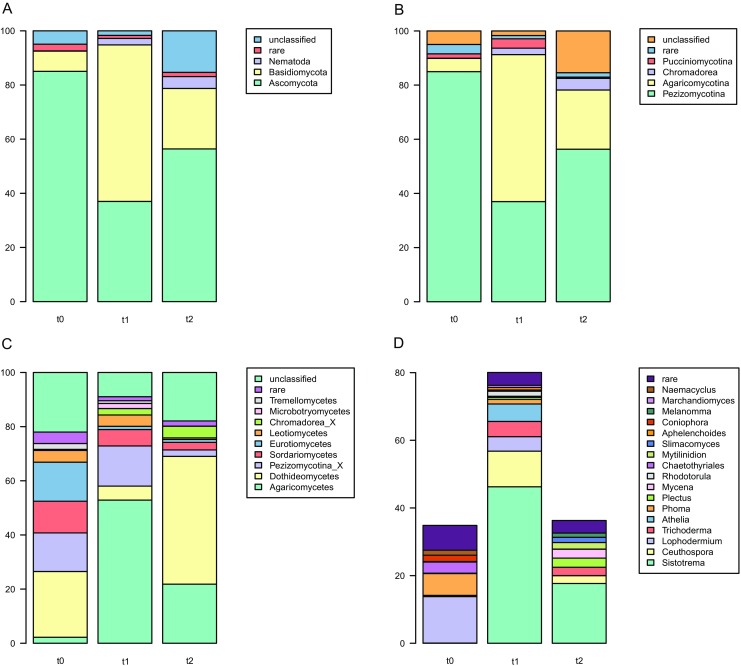
Classification of eukaryotic reads. Percentages of classified reads are shown at the following levels: subkingdoms (**a**), phyla (**b**), subclasses (**c**), and genera (**d**)

Eukaryotic libraries differed in biological replicates, similarly to bacterial ones. The libraries were dominated by fungal reads (Ascomycota 37–85% and Basidiomycota 7.5–57.8%) with minor quantities of Nematoda (Chromadorea, up to 4.4%) at later stages of decomposition (Fig. 7a). Ascomycotal reads prevailed in the libraries derived from fresh litter, then Basidiomycota were the most numerous in *t*_1_ samples and finally Ascomycota levels increased to ~ 65% in *t*_2_ litter. Each sample type harbored unique eukaryotic community at the genus level (Fig. 7b), in case of *t*_0_ ones hallmarked by *Lophodermium* and *Phoma*; *Sistotrema*, *Ceuthospora* (*Phacidium*), *Trichoderma*, and *Athelia* being characteristic for *t*_1_; and *Plectus* (nematode), *Mycena*, as well as *Mytilinidion* being typical for *t*_2_. However, due to relatively short 18S rRNA gene fragments being sequenced, only ~ 35 to 80% of reads could be classified down to this level.

### Metabolic Capabilities of Bacterial Community Are Different at Different Stages of Decomposition

PICRUSt analysis was performed to reveal potential functions encoded in genomes of bacteria whose 16S rRNA gene fragments were sequenced in our study. Nearest Sequenced Taxon Index (NSTI) analysis indicated that in most cases, a sequenced genome from the same genus could be found (NSTI < 0.05).

**Fig. 8:**
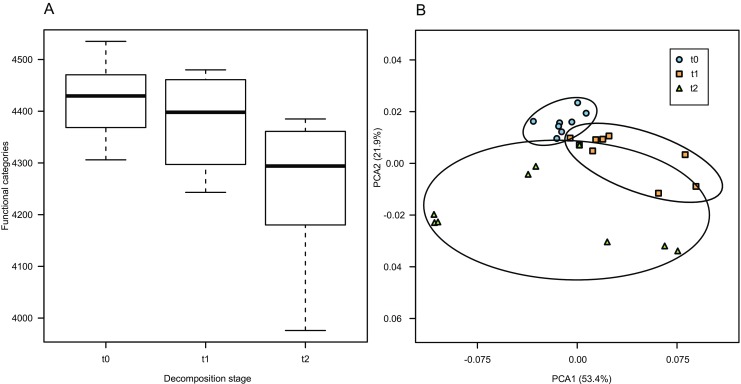
Diversity of functions encoded by bacterial genomes present in litter samples (whiskers denote standard error of the mean (SEM), **a**), nMDS analysis of Bray-Curtis distance matrix obtained from functional categories matrix (**b**). Stress: 0.187

**Fig. 9:**
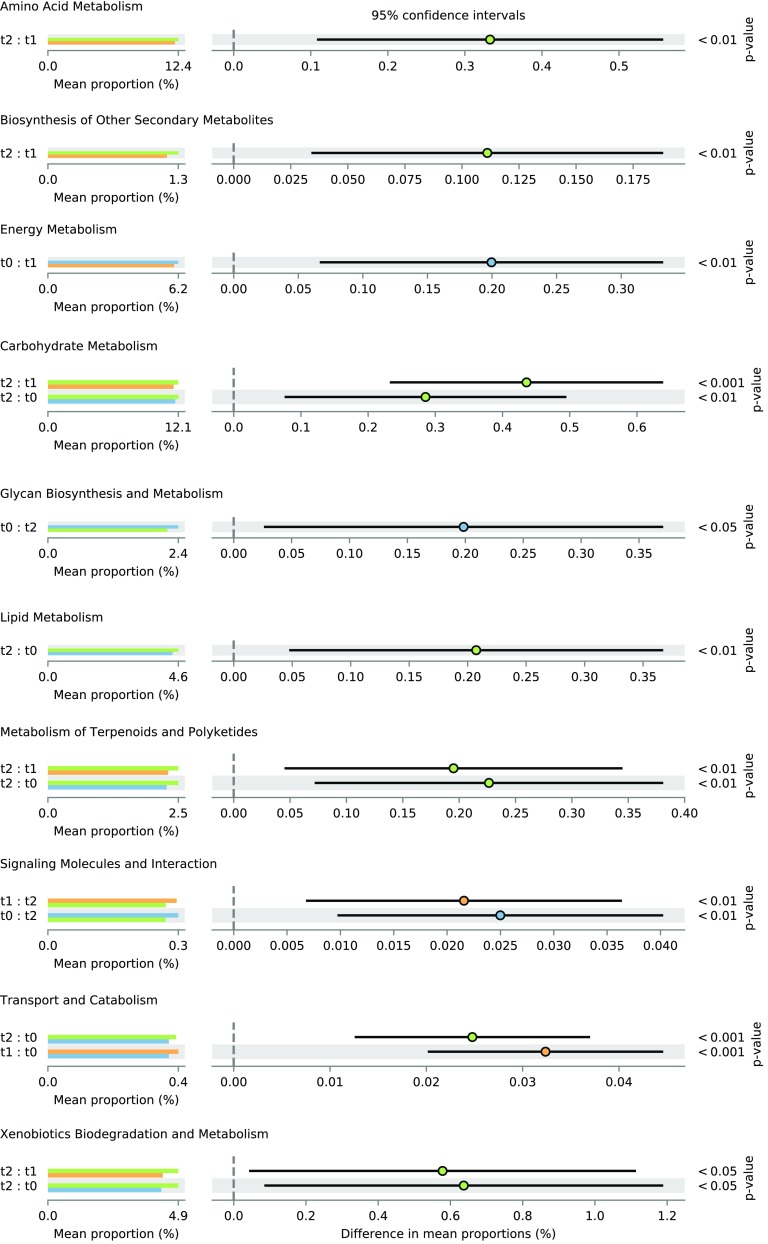
Selected functional categories of significantly different abundance. Dot and whiskers plots to the right of the barplots show 95% confidence intervals on the difference between respective samples, dot location indicates the mean, and the color of a given dot shows in which sample the proportion was higher. Bonferroni-corrected *P* value is given to the right of dot and whiskers plots

Overall diversity of functions was lower in the *t*_2_ samples than in *t*_1_ and *t*_0_ (*P* < 0.01; Fig. 8a). PCA analysis showed that functional composition of metagenomes was different in *t*_0_, *t*_1_, and *t*_2_ (Fig. 8b; *P* < 0.05, AMOVA); however, this effect was partially due to differences in variance (*P* < 0.01, PERMDISP). The pathways whose shares differed significantly between various sample types belonged mainly to “Metabolism” supercategory, but there were also “Signaling Molecules and Interaction” as well as “Transport and Catabolism” (Fig. 9).

Carbohydrate, amino acid, energy, lipid, xenobiotic, and terpenoid metabolism were the most frequent categories differing significantly. Genes encoding proteins participating in carbohydrates, terpenoids and xenobiotic metabolism were predicted to be more frequent in *t*_2_ communities than in the other two, while those engaged in lipid metabolism were estimated to be more frequent in *t*_2_ than in *t*_1_ only. Only the genes involved in energy metabolism were more probable to be encoded by organisms forming *t*_0_ community.

At the level of individual genes, these involved in various sugar transport (PTS system) and certain engaged in metabolism (e.g., sugar kinases, epimerases) as well as regulation (sugar utilization operon regulatory proteins) were predicted to be most abundantly represented in genomes of organisms from *t*_0_ samples, virtually absent from *t*_2_ samples with *t*_1_ in the middle. The same applies to other genes involved in import and utilization of soluble substrates such as amino acids, lipids, or amines, while β-glucosidases, potentially engaged in cellulose degradation, were predicted to be most frequent in *t*_1_ samples. In contrast, genes involved in biosynthesis of vitamins and cofactors, degradation of aromatic compounds (oxygenases and oxidases responsible for degradation of sterols and phenolic compounds such as catechol, 2,4-dichlorophenoxyacetate or 4-hydroxyacetophenone), as well as Fe^3+^ transporting ATPases were predicted to be most frequent in genomes of organisms dwelling in *t*_2_ samples.

## Discussion

The short time span of our experiment together with the small number of sampling time points causes our results to be rather a short sequence of snapshots than a time course of microbial succession on litter. Therefore, we avoid interpreting the results as “trends” and concentrate on differences between pairs of time points. Nevertheless, we think that the results provide an interesting insight into early stages of pine litter decomposition at the molecular level.

We observed no influence of enzymatic treatment neither on the amount of DNA isolated nor on microbial diversity. This might have been caused by the effectiveness of bead beating, making the action of enzymes pointless. In our previous study, we observed the influence of enzymes, as the method of isolation was based on less effective thermal/chemical lysis [61]. However, an alternative explanation is possible, assuming that cell envelopes of microbes in our samples were not digested by the enzymes used. Albeit possible, it is unlikely at least in the case of lysozyme, taking into consideration high numbers of Proteobacterial reads generated from the samples. We found that the enzyme worked well on *E. coli* DH10B in the buffer used for digestion. However, no increase in fungal DNA yield could be an effect of digestion conditions that were suboptimal for the chitinase used (according to the producer the optimal pH is 4, while the buffer used for digestion had pH 8). It seems that there is no reason for enzymatic pre-treatment of litter samples prior to bead-beating-based DNA isolation. Other studies report significant influence of lysozyme digestion on soil DNA yield, even in combination with bead-beating [62]; however, the bead-beating method was not as effective as in contemporary kits using dedicated bead-beaters.

DNA content of litter samples significantly increased during the studied decomposition period. This increase was caused by greater numbers of bacterial and fungal cells, as we found that pine DNA was almost completely degraded in *t*_1_ samples. This was confirmed by results of qPCR analysis of bacterial 16S rRNA and fungal ITS copy numbers. The number of bacterial sequences increased first (in the *t*_1_ samples), supporting our hypothesis that fresh litter is first colonized by bacteria, albeit, alternatively, this increase might have been caused by growth of phyllospheric organisms. It seems plausible that bacteria are responsible for pine DNA degradation.

We have found no archaeal reads in our libraries, which might mean that there were no Archaea in our samples. This is supported by results of a metaproteomic study concerning beech litter decomposition [16]. Nevertheless, it is possible that Archaea in pine litter might belong to a group whose 16S rRNA sequences differ from those amplifiable with the primer system we used, e.g., Nanoarchaeota, Altiarchaeota, or Diapherotrites or, as in all phyla the coverage was well below 90%, they could be members of generally amplifiable groups having non-amplifiable variants. There are reports of Archaea being cultured from Scots pine ectomycorrhizas (*Methanolobus*, *Halobacterium*, and unknown member of 11.c Crenarchaeota) [63], and ~ 2% of sequenced transcripts from spruce litter were coming from Archaea [26] and both facts support the latter possibility.

Shannon’s diversity, evenness, and species richness were consistently higher for bacterial communities than for fungal ones, which might be caused by the number of bacterial taxa being around one order of magnitude greater than the number of fungal ones in soils [64]. Moreover, bacterial diversity grew over time, while fungal communities became less diverse. This effect might have been caused by three factors: litter humidity, temperature, and time (decomposition stage). Although precipitation was lower in the *t*_0_–*t*_1_ period than in *t*_1_–*t*_2_ one, water content was the highest in *t*_1_ samples, which was probably caused by low temperature. During the initial period of the experiment (*t*_0_–*t*_1_), temperature was ~ 0 °C with occasional snow, and diversity did not change significantly, neither in the case of fungi, nor bacteria, which suggests weak selection. Later, warm period was hallmarked by bacterial species richness increase, showing possible colonization of litter by bacteria from the surrounding environment. Evenness was lower in *t*_2_ samples causing diversity to stay at the *t*_0_ level. In the case of eukaryotic community diversity, species richness and evenness decreased significantly in the *t*_2_ samples, suggesting strong selection.

*t*_0_ samples harbored a bacterial community similar to pine phyllosphere community [65], with high shares of Proteobacterial sequences, particularly members of the Acetobacteraceae and Pseudomonadaceae families being the hallmarks of phyllosphere. Differences might have resulted from tree species being different (Scots pine vs. limber pine), geographical distance (Central Europe vs. California), and physiological state of needles (dry, fallen vs. fresh ones). The eukaryotic *t*_0_ community consisted of Fungi, out of which Ascomycotal class Pezizomycotina prevailed, which was similar to fresh pine needles community studied by Millberg and colleagues [66] and unlike in senescent oak leaves, where Dothideomycetes prevailed [17]. Again, the differences might have resulted from geographical distance (Sweden vs. Poland) and physiological state of the needles. Eukaryotic sequences that could be identified down to the genus level belonged mainly to known phyllospheric fungi, such as *Phoma*, *Trichoderma*, or *Lophodermium*, and the latter might act as an early decomposer in litter [67, 68]. Low number of sequences was assigned to *Naemacyclus* (Helotiales, synonym *Cyclaneusma*), a genus comprising fungi causing needle cast [69]; thus, it is plausible that certain amount of collected needles were shed prematurely due to its action.

Differences between *t*_1_ and *t*_0_ samples were profound, in spite of temperature being below 0 °C most of the time. After 3 months of field incubation, the samples harbored communities that were drastically different from *t*_0_ ones; this fact demonstrates that colonization of fresh litter by soil and older litter-inhabiting organisms is rapid, regardless of harsh environmental conditions. This is in line with results of earlier studies on mass loss, e.g., [70] and with reports concerning decomposition of broadleaf litter [17, 19, 20]. Reads coming from phyllospheric organisms were less abundant than in the t_0_ samples (e.g., members of Acetobacteraceae, as well as fungi *Lophodermium* and *Phoma*), showing that, in spite of the lack of diversity decrease, selection operates at this stage of litter decomposition, but organisms unable to survive are replaced by colonizers. Obvious fungal colonizers were members of *Sistotrema*, ectomycorrhizal/saprophytic fungi of the Cantarellales order (Basidiomycota, Agaricomycetes), and *Ceuthospora* (synonym *Phacidium*, of Helotiales (Ascomycota, Dothideomycetes), [71], a genus comprising plant pathogenic fungi causing various diseases from needles rot in conifers to fruit rot in cranberries, apples, or pears [72, 73]. Reads derived from these organisms were virtually absent from *t*_0_ samples and constituted over 50% of all eukaryotic reads in *t*_1_ ones. Small numbers of reads obtained from *t*_0_ samples might be explained by spores sedimenting from air on the collected needles. The eukaryotic community at this stage of decomposition was more similar to the initial one than the bacterial one, suggesting that bacteria were able to grow more actively under winter conditions. C:N ratio was highest in *t*_0_ samples, indicating nitrogen depletion, probably due to organic nitrogen being consumed by microbes.

To no surprise, at the end of the experiment, the samples harbored communities different from both *t*_0_ and *t*_1_ ones. As seasonal differences in microbial community composition in spruce litter were not found to be large [26], we suggest that the differences visible in our data should be attributed to decomposition stage. Phyllospheric organisms were even less abundant in *t*_2_ than in *t*_1_, typical soil organisms could be found, such as nematodes of the Chromadorea class or bacteria of the *Rhizobium* genus, and the litter was colonized by organisms belonging to Dothideomycetes (Fungi, Ascomycota) and Sphingomonadaceae as well Bradyrhizobiaceae (Bacteria, Alphaproteobacteria), probably capable of degradation of recalcitrant compounds (lignocellulose, lignin) prevailing at this stage of decomposition [74]. The presence of nematodes might be one of the causes of slower increase of bacterial 16S rDNA counts, as the most abundant phylotype of this group (*Plectus*) is a bacterivore. This is supported by the results of co-correspondence analysis, as the influence of eukaryotic community on the bacterial one is significant and explains ~ 10% of variation. On the other hand, interactions among Eukaryota cannot be excluded, e.g., certain fungivorous nematodes most abundant in the *t*_1_ samples (e.g., members of the *Aphelenchoides* genus [75]) might be involved in much lower *Sistotrema* abundance in the *t*_2_ samples. The influence of bacteria on the eukaryotic community was even stronger (~ 22%). It might be explained, e.g., by providing nitrogen (by N_2_ fixation) that seems to be a limiting factor, particularly in *t*_0_ samples. This is confirmed by the results of CCA, wherein C/N was identified as a significant environmental variable. It is also possible that bacteria provided fungi with phosphorus, another limiting nutrient, by solubilizing its soil resources [76]. As the measured environmental variables were responsible for explaining of over 37% of variance, it seems plausible that they were the key drivers of microbial community structure changes.

Bacterial community structure at the level of genus was surprisingly similar to this found at the corresponding stages of decomposition in oak leaf litter [77], where *Pseudomonas*, *Sphingomonas*, and *Burkholderia* were among the dominating genera. Differences were mainly quantitative and were more pronounced in the phyllospheric communities. The most remarkable difference was the lack of *Duganella* (Oxalobacteriaceae, Betaproteobacteria) and *Frigoribacterium* (Micrococcaceae, Actinobacteria) in pine needles; these genera were replaced by unclassified members of the Acetobacteraceae family. Subsequent stages of pine litter decomposition harbored less Pedobacter. This fact suggests that, while plants assemble unique phyllospheric communities, decomposers’ assemblages, at least bacterial, may be more generic and similar regardless of the quality of litter.

Fungal communities decomposing pine litter are less similar to those degrading oak leaf litter described by Voriskova and colleagues [17]; however, general picture looks similar: phyllospheric communities are rapidly replaced by distinct communities characteristic for particular stages of decomposition. Certain fungi are abundant both in oak and pine decomposing litter, e.g., *Athelia*, *Rhodotorula*, and *Sistotrema*. Interestingly, *Sistotrema*, most abundant in 12-month-old oak litter [17], displays abundance peak in *t*_1_ samples. Thus, it is possible that fungal succession on decomposing pine litter “overtakes” the one on oak leaves.

A model of litter decomposition assumes that soluble compounds are degraded first, then degradation of hemicelluloses follows and the last stages comprise degradation of cellulose-lignin complex leading to increasing lignin/cellulose ratio [78]. We used PICRUSt to model possible metagenomes of decomposing litter to learn if the probable gene content supports this model.

However, one should bear in mind that the results obtained with PICRUSt are only an approximation of the “true” metagenome, and many factors, such as all biases influencing the underlying 16S rRNA gene fragment sequencing or differences in gene content of closely related organisms due to lateral gene transfer, may skew the results. Moreover, the set of functions derived from PICRUSt is only potential one, i.e., we do not know if the functions are actually expressed. Another limitation is that, due to the lack of a eukaryotic database, it was not possible to perform such an analysis for eukaryotic sequences. Therefore, the results should be treated with caution.

We expected a shift in community composition from organisms capable of various carbohydrates utilization to cellulose-degrading specialists, to lignin degraders, although the latter only to some extent, as our experiment concentrated on the first 8 months of litter decomposition. Our results support this view, as cellulolytic genes (β-glucosidases) were most abundant in *t*_2_ samples, and certain genes that might be involved in lignin degradation (oxygenases and oxidases) were also found. The genes engaged in active transport of cellobiose (cellulose degradation product) into the cell (PTS system) were most frequent in *t*_0_ samples and permeases allowing for passive import of this sugar were most numerous in *t*_1_ samples. This picture might mean that during the initial stage of litter decomposition (*t*_0_) after depletion of other easily accessible sugars, active transfer of cellobiose is required, most probably due to the low concentration of this compound; then, fungi start producing cellulases causing increase of the cellobiose concentration, which allows passive transfer; and finally, bacterial cellulolytic activity may supplement fungal activity (*t*_2_). The latter claim was partially supported by results of Zifcakova and colleagues, who found that Fungi were responsible for only half of carbohydrates metabolism-involved transcripts production in spruce litter [26]. Given the importance of coniferous litter decomposition and taking into account all the limitations of the methodology as well as the general scarcity of molecular studies devoted to coniferous litter decomposition, more work is needed to understand which organisms are responsible for particular processes during decomposition.

## Conclusions

There is no need to supplement contemporary bead beating-based kits with cell wall-degrading enzymes. Bacterial and fungal diversity changes differently during initial stages of decomposition. Microbial succession on decomposing pine litter is rapid and initial phyllospheric communities are replaced with distinct assemblage characteristic for particular stages of decomposition. Changes in communities seem to be driven not only by physicochemical variables of litter but also by interactions among bacteria, fungi, and nematodes.

## Electronic Supplementary Material

ESM 1 (ODT 67 kb)

## References

[1] FAO (2010) Global forest resources assessment 2010. http://www.fao.org/forest-resources-assessment/en/

[2] Bradford MA, Berg B, Maynard DS, Wieder WR, Wood SA (2016). Understanding the dominant controls on litter decomposition. J. Ecol..

[3] Berg B, Laskowski R (2006) Litter decomposition: a guide to carbon and nutrient turnover. Elsevier

[4] David JF (2014). The role of litter-feeding macroarthropods in decomposition processes: a reappraisal of common views. Soil Biol. Biochem..

[5] Persson T, Bååth E, Clarholm M (1980). Trophic structure, biomass dynamics and carbon metabolism of soil organisms in a Scots pine forest. Oikos.

[6] Berg B, Hannus K, Popoff T, Theander O (1982). Changes in organic chemical components of needle litter during decomposition. Long-term decomposition in a Scots pine forest. I. Can. J. Bot..

[7] Staaf H, Berg B (1982). Accumulation and release of plant nutrients in decomposing Scots pine litter. Long-term decomposition in a Scots pine forest. II. Can. J. Bot..

[8] Berg B (1986). Nutrient release from litter and humus in coniferous forest soils—a mini review. Scand. J. For. Res..

[9] Berg B (1988). Dynamics of nitrogen (15N) in decomposing Scots pine (*Pinus sylvestris*) needle litter. Long-term decomposition in a Scots pine forest. VI. Can. J. Bot..

[10] Bardgett RD (2005) The biology of soil: a community and ecosystem approach. Oxford University Press

[11] Cepáková Š, Frouz J (2015). Changes in chemical composition of litter during decomposition: a review of published ^13^C NMR spectra. J. Soil Sci. Plant Nutr..

[12] Ponge J-FF (1991). Succession of fungi and fauna during decomposition of needles in a small area of Scots pine litter. Plant Soil.

[13] Tokumasu S, Aoki T, Oberwinkler F (1994). Fungal succession on pine needles in Germany. Mycoscience.

[14] Berg MP, Kniese J, Bedaux J, Verhoef H (1998). Dynamics and stratification of bacteria and fungi in the organic layers of a scots pine forest soil. Biol. Fertil. Soils.

[15] Aneja MK, Sharma S, Fleischmann F, Stich S, Heller W, Bahnweg G, Munch JC, Schloter M (2006). Microbial colonization of beech and spruce litter—influence of decomposition site and plant litter species on the diversity of microbial community. Microb. Ecol..

[16] Schneider T, Keiblinger KM, Schmid E, Sterflinger-Gleixner K, Ellersdorfer G, Roschitzki B, Richter A, Eberl L, Zechmeister-Boltenstern S, Riedel K (2012). Who is who in litter decomposition? Metaproteomics reveals major microbial players and their biogeochemical functions. ISME J..

[17] Voříšková J, Baldrian P (2013). Fungal community on decomposing leaf litter undergoes rapid successional changes. ISME J..

[18] Urbanová M, Šnajdr J, Baldrian P (2015). Composition of fungal and bacterial communities in forest litter and soil is largely determined by dominant trees. Soil Biol. Biochem..

[19] Tláskal V, Voříšková J, Baldrian P (2016). Bacterial succession on decomposing leaf litter exhibits a specific occurrence pattern of cellulolytic taxa and potential decomposers of fungal mycelia. FEMS Microbiol. Ecol..

[20] Purahong W, Wubet T, Lentendu G, Schloter M, Pecyna MJ, Kapturska D, Hofrichter M, Krüger D, Buscot F (2016). Life in leaf litter: novel insights into community dynamics of bacteria and fungi during litter decomposition. Mol. Ecol..

[21] Dilly O, Munch J-C (1996). Microbial biomass content, basal respiration and enzyme activities during the course of decomposition of leaf litter in a black alder (*Alnus glutinosa* (L.) Gaertn.) forest. Soil Biol. Biochem..

[22] Baldrian P (2017). Forest microbiome: diversity, complexity, and dynamics. FEMS Microbiol. Rev..

[23] Swift MJ, Heal OW, Anderson JM (1979) Decomposition in terrestrial ecosystems. University of California Press

[24] Melillo JM, Aber JD, Linkins AE, Ricca A, Fry B, Nadelhoffer KJ (1989). Carbon and nitrogen dynamics along the decay continuum: plant litter to soil organic matter. Plant Soil.

[25] Baldrian P, Kolařík M, Štursová M, Kopecký J, Valášková V, Větrovský T, Žifčáková L, Šnajdr J, Rídl J, Vlček Č, Voříšková J (2012). Active and total microbial communities in forest soil are largely different and highly stratified during decomposition. ISME J.

[26] Žifčáková L, Větrovský T, Howe A, Baldrian P (2016). Microbial activity in forest soil reflects the changes in ecosystem properties between summer and winter. Environ. Microbiol..

[27] Osono T (2006). Role of phyllosphere fungi of forest trees in the development of decomposer fungal communities and decomposition processes of leaf litter. Can. J. Microbiol..

[28] Voříšková J, Brabcová V, Cajthaml T, Baldrian P (2014). Seasonal dynamics of fungal communities in a temperate oak forest soil. New Phytol..

[29] Ward DM, Weller R, Bateson MM (1990). 16S rRNA sequences reveal numerous uncultured microorganisms in a natural community. Nature.

[30] Hugenholtz P, Goebel BM, Pace NR (1998) Impact of culture independent studies on the emerging phylogenetic view of bacterial diversity. J. Bacteriol. 180: 4765–4774. doi: 0021–9193/98/$04.00+0 10.1128/jb.180.18.4765-4774.1998 PMC107498 9733676

[31] Steele HL, Streit WR (2005). Metagenomics: advances in ecology and biotechnology. FEMS Microbiol. Lett..

[32] Handelsman J, Rondon MR, Brady SF, Clardy J, Goodman RM (1998). Molecular biological access to the chemistry of unknown soil microbes: a new frontier for natural products. Chem. Biol..

[33] Rondon MR, Bettermann AD, Brady SF (2000). Cloning the soil metagenome: a strategy for accessing the genetic and functional diversity of uncultured microorganisms. Appl. Environ. Microbiol..

[34] Štursová M, Žifčáková L, Leigh MB, Burgess R, Baldrian P (2012). Cellulose utilization in forest litter and soil: identification of bacterial and fungal decomposers. FEMS Microbiol. Ecol..

[35] Tsai Y-L, Olson BH (1992). Detection of low numbers of bacterial cells in soils and sediments by polymerase chain reaction. Apllied Environ. Microbiol..

[36] Quast C, Pruesse E, Yilmaz P, Gerken J, Schweer T, Yarza P, Peplies J, Glöckner FO (2013). The SILVA ribosomal RNA gene database project: improved data processing and web-based tools. Nucleic Acids Res..

[37] Klindworth A, Pruesse E, Schweer T, Peplies J, Quast C, Horn M, Glöckner FO (2013). Evaluation of general 16S ribosomal RNA gene PCR primers for classical and next-generation sequencing-based diversity studies. Nucleic Acids Res..

[38] IDT (2014) IDT Oligo Analyzer. http://eu.idtdna.com/calc/analyzer

[39] Faircloth BC, Glenn TC (2012). Not all sequence tags are created equal: designing and validating sequence identification tags robust to indels. PLoS One.

[40] Schloss PD, Westcott SL, Ryabin T, Hall JR, Hartmann M, Hollister EB, Lesniewski RA, Oakley BB, Parks DH, Robinson CJ, Sahl JW, Stres B, Thallinger GG, van Horn DJ, Weber CF (2009). Introducing mothur: open-source, platform-independent, community-supported software for describing and comparing microbial communities. Appl. Environ. Microbiol..

[41] Gołębiewski M, Deja-Sikora E, Cichosz M, Tretyn A, Wróbel B (2014). 16S rDNA pyrosequencing analysis of bacterial community in heavy metals polluted soils. Microb. Ecol..

[42] Gołębiewski M, Całkiewicz J, Creer S, Piwosz K (2017). Tideless estuaries in brackish seas as possible freshwater-marine transition zones for bacteria: the case study of the Vistula river estuary. Environ. Microbiol. Rep..

[43] Quince C, Lanzen A, Davenport RJ, Turnbaugh PJ (2011). Removing noise from pyrosequenced amplicons. BMC Bioinformatics.

[44] Huse SM, Welch DM, Morrison HG, Sogin ML (2010). Ironing out the wrinkles in the rare biosphere through improved OTU clustering. Environ. Microbiol..

[45] Edgar RC, Haas BJ, Clemente JC, Quince C, Knight R (2011). UCHIME improves sensitivity and speed of chimera detection. Bioinformatics.

[46] Wang Q, Garrity GM, Tiedje JM, Cole JR (2007). Naive Bayesian classifier for rapid assignment of rRNA sequences into the new bacterial taxonomy. Appl. Environ. Microbiol..

[47] Guillou L, Bachar D, Audic S, Bass D, Berney C, Bittner L, Boutte C, Burgaud G, de Vargas C, Decelle J, del Campo J, Dolan JR, Dunthorn M, Edvardsen B, Holzmann M, Kooistra WHCF, Lara E, le Bescot N, Logares R, Mahé F, Massana R, Montresor M, Morard R, Not F, Pawlowski J, Probert I, Sauvadet AL, Siano R, Stoeck T, Vaulot D, Zimmermann P, Christen R (2013). The Protist Ribosomal Reference database (PR2): a catalog of unicellular eukaryote small sub-unit rRNA sequences with curated taxonomy. Nucleic Acids Res..

[48] Sheneman L, Evans J, Foster JA (2006). Clearcut: a fast implementation of relaxed neighbor joining. Bioinformatics.

[49] Lozupone C, Knight R (2005). UniFrac: a new phylogenetic method for comparing microbial communities. Appl. Environ. Microbiol..

[50] Horn HS (1966). Measurement of “overlap” in comparative ecological studies. Am. Nat..

[51] Bray RJ, Curtis JT (1957). An ordination of the upland forest communities of southern Winsconin. Ecol. Monogr..

[52] Oksanen J, Blanchet FG, Kindt R, et al (2013) Vegan: community ecology package. R package, https://cran.r-project.org/web/packages/vegan/index.html

[53] ter Braak CJF, Schaffers AP (2004). Co-correspondence analysis: a new ordination method to relate two community compositions. Ecology.

[54] Simpson GL (2009) Cocorresp: co-correspondence analysis ordination methods. R package, https://github.com/gavinsimpson/cocorresp

[55] Langille M, Zaneveld J, Caporaso JG (2013). Predictive functional profiling of microbial communities using 16S rRNA marker gene sequences. Nat. Biotechnol..

[56] DeSantis TZ, Hugenholtz P, Larsen N (2006). Greengenes, a chimera-checked 16S rRNA gene database and workbench compatible with ARB. Appl. Environ. Microbiol..

[57] Parks DH, Tyson GW, Hugenholtz P, Beiko RG (2014). STAMP: statistical analysis of taxonomic and functional profiles. Bioinformatics.

[58] R Core Team (2016) R: a language and environment for statistical computing. https://www.r-project.org/

[59] Harrell Jr FE, with contributions from Charles Dupont, many others. (2014) Hmisc: Harrell miscellaneous. R package, https://cran.r-project.org/web/packages/Hmisc/index.html

[60] McMurdie PJ, Holmes S (2013). Phyloseq: an R package for reproducible interactive analysis and graphics of microbiome census data. PLoS One.

[61] Deja-Sikora E (2012) Search for bacterial cadmium, zinc, lead, copper and chromium resistance genes in metagenome of soils polluted with heavy metals. Nicolaus Copernicus University

[62] Krsek M, Wellington EMH (1999). Comparison of different methods for the isolation and purification of total community DNA from soil. J. Microbiol. Methods.

[63] Bomberg M, Montonen L, Timonen S (2010). Anaerobic Eury- and Crenarchaeota inhabit ectomycorrhizas of boreal forest Scots pine. Eur. J. Soil Biol..

[64] Larsen BB, Miller EC, Rhodes MK, Wiens JJ (2017). Inordinate fondness multiplied and redistributed: the number of species on earth and the new pie of life. Q. Rev. Biol..

[65] Carrell AA, Frank AC (2014) *Pinus flexilis* and *Picea engelmannii* share a simple and consistent needle endophyte microbiota with a potential role in nitrogen fixation. Front. Microbiol. 5: . doi: 10.3389/fmicb.2014.00333 10.3389/fmicb.2014.00333 PMC4082182 25071746

[66] Millberg H, Boberg J, Stenlid J (2015). Changes in fungal community of Scots pine (*Pinus sylvestris*) needles along a latitudinal gradient in Sweden. Fungal Ecol..

[67] Boberg JB, Ihrmark K, Lindahl BD (2011). Decomposing capacity of fungi commonly detected in *Pinus sylvestris* needle litter. Fungal Ecol..

[68] Osono T, Hirose D (2011). Colonization and lignin decomposition of pine needle litter by *Lophodermium pinastri*. For. Pathol..

[69] van der PAS JB, Slater-hayes JD, Gadgil PD, Bulman L (1984) Cyclaneusma (Naemacyclus) needle-cast of *Pinus radiata* in New Zealand. 2: reduction in growth of the host, and its economic implication. New Zeal J For Sci 14:197–209

[70] Vestgarden L (2001). Carbon and nitrogen turnover in the early stage of Scots pine (*Pinus sylvestris* L.) needle litter decomposition: effects of internal and external nitrogen. Soil Biol. Biochem..

[71] Crous PW, Quaedvlieg W, Hansen K, Hawksworth DL, Groenewald JZ (2014). *Phacidium* and *Ceuthospora* (Phacidiaceae) are congeneric: taxonomic and nomenclatural implications. IMA Fungus.

[72] Farr DF, Rossman AY (2018) Fungal databases, U.S. National Fungus Collecsions, ARS, USDA. https://nt.ars-grin.gov/fungaldatabases. Accessed 1 Feb 2018

[73] Wiseman MS, Kim YK, Dugan FM, Rogers JD, Xiao CL (2016). A new postharvest fruit rot in apple and pear caused by *Phacidium lacerum*. Plant Dis..

[74] Berg B, McClaugherty C (2003). Plant litter. Decomposition, humus formation, carbon sequestration.

[75] Bae Y-S, Knudsen GR (2001). Influence of a fungus-feeding nematode on growth and biocontrol efficacy of *Trichoderma harzianum*. Phytopathology.

[76] Taktek S, Trépanier M, Servin PM, St-Arnaud M, Piché Y, Fortin JA, Antoun H (2015). Trapping of phosphate solubilizing bacteria on hyphae of the arbuscular mycorrhizal fungus *Rhizophagus irregularis* DAOM 197198. Soil Biol. Biochem..

[77] Baldrian P, Zrůstová P, Tláskal V, Davidová A, Merhautová V, Vrška T (2016). Fungi associated with decomposing deadwood in a natural beech-dominated forest. Fungal Ecol..

[78] Berg B, Matzner E (1997). Effect of N deposition on decomposition of plant litter and soil organic matter in forest systems. Environ. Rev..

[79] White TJ, Bruns T, Lee S, Taylor J (1990) Amplification and direct sequencing of fungal ribosomal RNA genes for phylogenetics. In: PCR protocols: a guide to methods and applications. pp 315–322

[80] Wakelin SA, Colloff MJ, Harvey PR, Marschner P, Gregg AL, Rogers SL (2007). The effects of stubble retention and nitrogen application on soil microbial community structure and functional gene abundance under irrigated maize. FEMS Microbiol. Ecol..

[81] Neefs J-M, Van de Peer Y, De Rijk P (1993). Compilation of small ribosomal subunit RNA structures. Nucleic Acids Res..

[82] Hadziavdic K, Lekang K, Lanzen A, Jonassen I, Thompson EM, Troedsson C (2014). Characterization of the 18S rRNA gene for designing universal eukaryote specific primers. PLoS One.

