# Supplementary material for: Rapid Microbial Community Changes During Initial Stages of Pine Litter Decomposition
Source: Microb Ecol. 2018 May 30;77(1):56–75. doi: 10.1007/s00248-018-1209-x (PMC6318262; doi:10.1007/s00248-018-1209-x)
Supplement: Supplementary file 1 [file 248_2018_1209_MOESM1_ESM.odt]

Fig. 1: Climatic data for Sierbowice site. Daily means from six nearby stations were averaged to yield the presented values. Temperature (degrees Celsius)  – A, precipitation (mm) – B, snowcover (mm) – C. Sampling times are marked with vertical discontigious lines  

Fig. 2: CCA analysis of bacterial (left hand side) and eukaryotic (right hand site) communities. Only significant variables (p<0.01) wre plotted. fresh – t0 samples, 3m – t1 samples, 6m – t2 samples, red crosses denote species

Fig. 3: CoCA analysis, plot of sites. Bacterial communities on left hand side, eukaryotic ones on right hand side. The communities were displayed on two panels to avoid crowded plot. The smaller the distance between respective communities (shown on two panels), the greater influence they exert on each other, e.g. for 6mI1 the distance is small, while for 3mF3 it is large. fresh – t0 samples, 3m – t1 samples, 6m – t2 samples
